# A Prospective Comparison of Subjective Symptoms and Neurophysiological Findings in the Assessment of Neuropathy in Cancer Patients

**DOI:** 10.3390/diagnostics14242861

**Published:** 2024-12-19

**Authors:** Vera Elisabeth Adreana Kleinveld, Miriam Emmelheinz, Daniel Egle, Magdalena Ritter, Wolfgang N. Löscher, Christian Marth, Corinne Gosewina Cornelia Horlings, Julia Wanschitz, Christine Brunner

**Affiliations:** 1 Department of Neurology, Medical University Innsbruck, 6020 Innsbruck, Austria; elisabeth.kleinveld@tirol-kliniken.at (V.E.A.K.); wolfgang.loescher@tirol-kliniken.at (W.N.L.); corinne.horlings@tirol-kliniken.at (C.G.C.H.); julia.wanschitz@tirol-kliniken.at (J.W.); 2 Department of Obstetrics and Gynecology, Medical University Innsbruck, 6020 Innsbruck, Austria; miriam.emmelheinz@tirol-kliniken.at (M.E.); daniel.egle@tirol-kliniken.at (D.E.); magdalena.ritter@dornbirn.at (M.R.); christian.marth@tirol-kliniken.at (C.M.)

**Keywords:** taxane-based chemotherapy-induced polyneuropathy, nerve conduction studies, toxicity, adverse events

## Abstract

**Objectives:** Neurotoxic effects causing peripheral nerve damage have been reported for several chemotherapy agents. There is no established and standardized method to assess the presence of chemotherapy-induced peripheral neuropathy (CIPN). We compared patient-reported CIPN symptoms to neurophysiological findings and neurological assessments in patients receiving taxane-based chemotherapy. **Methods:** Patients scheduled to receive taxane-based chemotherapy for the treatment of gynecologic cancer were included and prospectively followed for up to 9 months after chemotherapy discontinuation, between May 2020 and January 2023. Patient-reported symptoms, using the EORTC-QLQ-CIPN20 questionnaire, and nerve conduction studies (NCSs) were performed at baseline, halfway through the treatment cycle, at the end of the treatment, 3 months after treatment, and 6–9 months after treatment. **Results:** A total of 149 patients were included. Overall, 47.0% of patients reported symptoms compatible with CIPN at any of the follow-ups. Subjective symptoms did not correlate with nerve conduction studies. SNAP amplitudes at baseline were lower in patients who developed CIPN compared to the group without CIPN. **Conclusions:** The overall diagnostic accuracy of electrophysiological parameters as a marker for CIPN was low.

## 1. Introduction

Neurotoxic effects causing distal length-dependent motor and sensory nerve damage have been reported for several chemotherapy agents like taxanes and platinum derivatives [1]. Neurotoxicity depends on the total cumulative dose and the type of drug used [2]. Patients with chemotherapy-induced peripheral neuropathy (CIPN) experience symptoms which might lead to the modification or discontinuation of chemotherapy [3], potentially leading to sub-optimal treatment effects. It is important to identify and monitor CIPN repeatedly during chemotherapy treatment as it can occur at any time point during treatment. The reported CIPN prevalence during the first month of chemotherapy is as high as 68.1%, and up to 30% of patients continue to suffer from CIPN months to years after the discontinuation of chemotherapy [1,4]. In CIPN, sensory symptoms such as pain, numbness, paresthesia, and balance and gait disturbances predominate over motor and autonomic dysfunction [5]. The constellation of symptoms in CIPN is related to fatigue, anxiety, and physiological distress [6], interferes with quality of life [7], and can have a long-term impact on functional performance [4]. Early diagnosis enables proactive rehabilitation in patients with CIPN, which facilitates the management and improvement of CIPN-associated physical functioning deficits [8].

So far, there is no gold standard to diagnose CIPN, which might contribute to CIPN being underreported and underestimated by physicians [9,10,11]. While nerve conduction studies (NCSs) are considered a reliable method to diagnose and quantify polyneuropathies of various etiologies [12], they are uncomfortable and time-consuming, and previous studies have suggested that discrepancies between patient-reported outcomes and objective electrophysiological assessments may exist in CIPN [13,14,15,16].

To assess the value of NCSs in patients with and without symptoms of CIPN, there is a need for prospective longitudinal studies, which include pre-chemotherapy baseline data and multiple follow-ups during treatment. We assessed NCSs prospectively in a large cohort of patients undergoing taxane-based chemotherapy for the treatment of gynecologic cancer before, during, and after treatment.

## 2. Methods

### 2.1. Patients and Methods

Patients scheduled to receive taxane-based chemotherapy for the treatment of gynecologic cancer (breast, cervical, ovarian, endometrial, and vulvar) were included and prospectively followed at the University Hospital of Innsbruck, Austria, between May 2020 and January 2023. Patients with a previously diagnosed polyneuropathy grade 2 or higher were excluded.

Patients were recruited from the CROPSI study, a study on the efficacy of cryotherapy versus cryocompression in the prevention of upper limb CIPN in patients with gynecologic cancer, conducted between May 2020 and January 2023. All patients provided written informed consent in accordance with the Declaration of Helsinki. The study was approved by the local ethics committee and registered as a clinical trial with the following number: NCT04632797. 

Individuals received different chemotherapy protocols that comprised either taxane monotherapy and a taxane- and platinum-based chemotherapy or a taxane- and anthracycline-based chemotherapy (either taxane first, anthracycline first or both chemotherapy regimens simultaneously). 

Detailed neurophysiological and clinical assessments of the dominant upper extremity were performed, and patient-reported outcomes were derived at baseline (T0), halfway through the treatment cycle (T1), at the end of the treatment (T2), 3 months after treatment (T3) and 6-9 months after treatment (T4).

### 2.2. Patient-Reported Outcomes (PROs)

Patients filled in the European Organization for Research and Treatment of Cancer Chemotherapy-Induced Peripheral Neuropathy 20 (EORTC-QLQ-CIPN20) [17], a 20-item scale assessing sensory (9 items), motor (8 items) and autonomic (3 items) symptoms. In this self-reported scale, patients indicate the degree to which they have experienced symptoms indicative of CIPN during the past week. Symptoms are scored on a 4-point Likert scale from 1 (not at all) to 4 (very much), and a sum score was calculated. Since our study population included only female patients, question 20 on erectile dysfunction was excluded from our analysis. Higher sum scores represent higher levels of symptom burden. In this paper, use is made of data from PROFILES (Patient-Reported Outcomes Following Initial treatment and Long-term Survivorship) [18]. In this large cohort of healthy female participants, excluding the patients with a history of cancer and excluding question 20 on erectile dysfunction, an EORTC-QLQ-CIPN20 of 30 represents the 95th percentile. Also, others found a mean EORTC-QLQ-CIPN20 score of 29 or higher in paclitaxel/carboplatin-treated patients with CTCAE scores ≥1 [19]. An EORTC-QLQ-CIPN20 score ≥30 was considered clinically compatible with CIPN. Patients with symptoms of CIPN at baseline and patients without any data on the presence or absence of CIPN symptoms during follow-up were excluded.

### 2.3. Nerve Conduction Studies (NCSs)

For all electrophysiological examinations, a Dantec^®^ Keypoint^®^ (Natus, Middleton, WI, USA) was used. To reduce patient burden and given the symmetrical nature of CIPN, one side was measured. Generally, the dominant arm was measured, except for patients with a history of carpal tunnel syndrome or other pre-existing mononeuropathies on the dominant side. In that case, the other side was measured. Standardized motor and sensory NCSs were performed in accordance with our laboratory’s standard operating procedures and normative values. Motor nerve conduction studies included the median (m. APB) and ulnar nerve (m. ADM). Antidromic sensory nerve conduction studies included the radial, median and ulnar nerve recorded over the anatomical snuff box and from digitus II and V, respectively. The responses after supramaximal stimulation were measured from peak to peak for sensory nerve action potential (SNAP) amplitudes and baseline to peak for compound muscle action potential (CMAP) amplitudes. Latency, SNAP amplitude, CMAP amplitude and nerve conduction velocity were registered. 

### 2.4. Statistical Methods

Patient characteristics were summarized using descriptive statistics. The normality of the data was assessed using Shapiro–Wilk tests and visually using histograms and Q-Q plots. Data are shown as mean and standard deviation (SD) unless stated otherwise. An independent samples *t*-test was used to compare parameters among two independent groups and Mann–Whitney U Test was used as a non-parametric alternative. For paired parameters, paired *t*-tests were used. Pearson’s chi-square was used to compare categorical variables. Correlation analysis was performed using Spearman’s rho for non-normally distributed data and Pearson’s for normally distributed data. For both PROs and NCSs, delta scores were calculated as the difference in score between baseline and respective follow-up. For electrophysiological parameters, the change from baseline was expressed as a percentage of change from baseline. For this, follow-up electrophysiological parameters were obtained from the follow-up visit with the highest EORTC-QLQ-CIPN20 score. If the highest EORTC-QLQ-CIPN20 score was equal in multiple follow-up visits, the values obtained at the first visit were included in the analysis. We excluded electrophysiological parameters with ≥100% change from baseline, as we assumed this was the result of a measuring error.

A two-tailed *p*-value below 0.05 was considered statistically significant. Statistical analysis was performed using SPSS version 29.0.0 (IBM Corp, Armonk, NY, USA) and GraphPad Prism version 9.4.1 for Windows (Graphpad Software, Boston, MA, USA, www.graphpad.com).

## 3. Results

149 patients were included. All patients were female. Patients were divided into two groups depending on whether they developed symptoms of CIPN at any time point during follow-up (EORTC-QLQ-CIPN20 score of ≥30) or not. The baseline demographic and clinical characteristics, for all patients as well as the two groups, were well balanced (see Table 1). Overall, 47.0% of patients developed symptoms of CIPN. The baseline nerve conduction study parameters can be found in Table 2.

The mean EORTC-QLQ-CIPN20 score at baseline was 21.9 (±2.6) for patients who developed CIPN symptoms and 21.1 (±2.6) for patients without CIPN symptoms. At follow-up, the mean EORTC-QLQ-CIPN20 in patients with CIPN symptoms was 38.8 (±8.0), which was significantly higher compared to baseline (*p* < 0.01). For patients without CIPN symptoms, the mean EORTC-QLQ-CIPN20 score at follow-up was 22.8 (±2.9), which was significantly higher compared to baseline as well (*p* < 0.01). 

**Figure 1:**
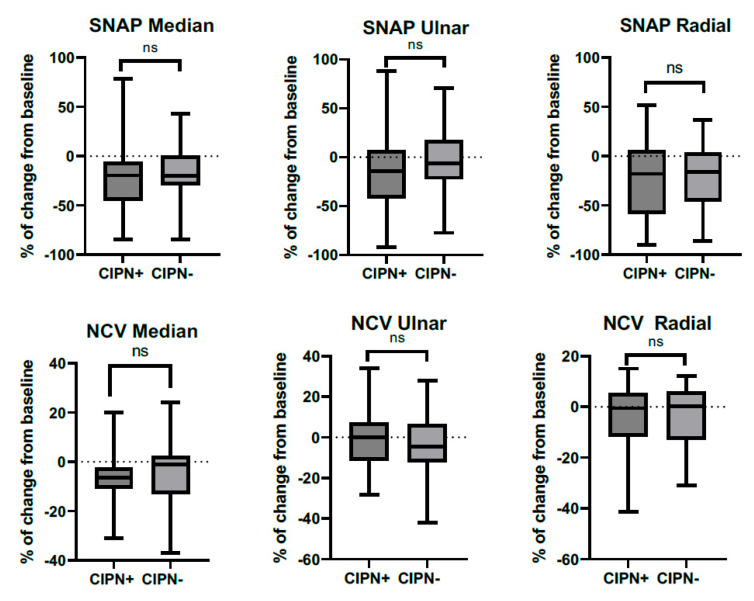
Longitudinal changes in sensory nerve action potentials and sensory nerve conduction velocities in patients with and without CIPN symptoms at baseline and follow-up. CIPN+: patients that reported an EORTC-QLQ-CIPN20 ≥30 at specific follow-up. Data from the follow-up visit with the highest EORTC-QLQ-CIPN20 score were used. Abbreviations: CIPN, chemotherapy-induced polyneuropathy; EORTC-QLQ-CIPN-20, European Organization for Research and Treatment of Cancer Chemotherapy-Induced Peripheral Neuropathy 20; NCV, nerve conduction velocity; SNAP, sensory nerve action potential.

**Figure 2:**
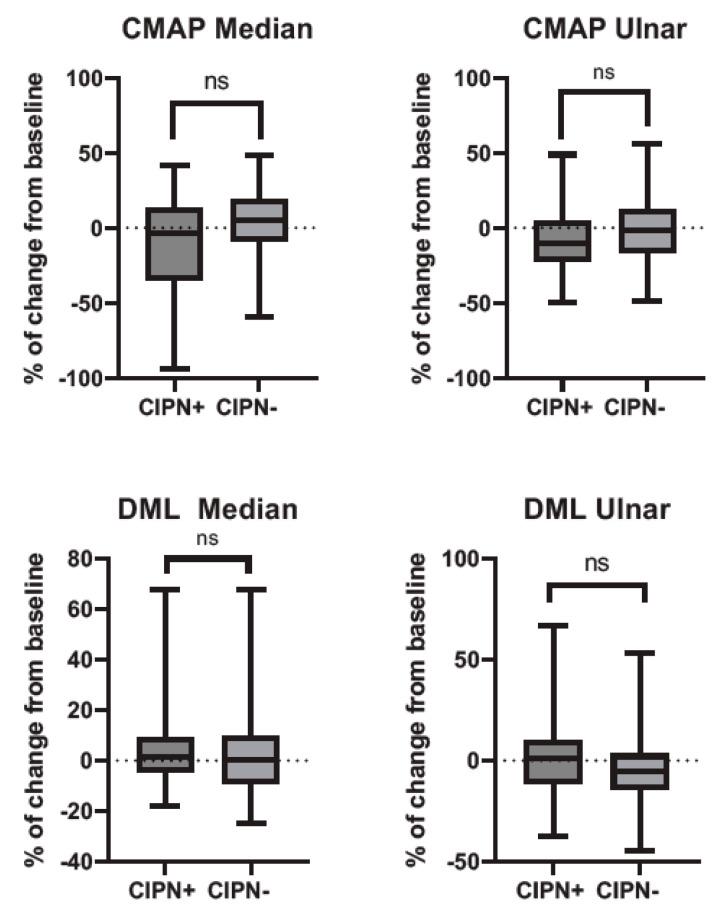
Longitudinal changes in motor nerve action potentials and distal motor latency in patients with and without CIPN symptoms at specific follow-ups. CIPN+: patients that reported an EORTC-QLQ-CIPN20 score ≥30 at specific follow-up. Abbreviations: CIPN, chemotherapy-induced polyneuropathy; CMAP, compound muscle action potential; DML, distal motor latency; EORTC-QLQ-CIPN-20, European Organization for Research and Treatment of Cancer Chemotherapy-Induced Peripheral Neuropathy 20.

The percentage of change from baseline of sensory nerve action potential (SNAP) amplitudes and nerve conduction velocities are displayed in Figure 1, while compound muscle action potential (CMAP) amplitudes and distal motor latencies are displayed in Figure 2. For all electrophysiological parameters, there was no significant difference in the % of change from baseline between the patients with and without symptoms of CIPN.

In both patients with and without CIPN symptoms, the percentage of change from baseline of electrophysiological parameters did not correlate with the percentage of change in self-reported symptoms (see Table 3). 

The ROC analyses of the performance of electrophysiological changes to discriminate between patients with and without CIPN resulted in AUC values of 0.391–0.572 (see Table 4). 

## 4. Discussion

In this cohort of patients with gynecological cancer, longitudinal data on the occurrence of CIPN symptoms, as well as serial NCSs, were obtained. Overall, 47.0% of patients developed subjective symptoms of CIPN. We were able to demonstrate considerable disparities between subjective patient-reported symptoms and nerve conduction studies.

Previous studies reported various prevalence rates of CIPN [1,4,20]. This might be explained by differences in the timing of assessments and differences in assessment modalities, as there currently is no gold standard for the diagnosis of CIPN. In this prospective study with multiple follow-ups during and after chemotherapy, we assume the occurrence of CIPN-related symptoms could be ascertained accurately. As patients were followed up after treatment cessation, those who deteriorated in the period after the last chemotherapy cycle (a phenomenon known as ‘coasting’) could also be detected [20].

NCSs upon follow-up revealed no differences in amplitude decrease, latency increase or conduction velocity decrease, comparing patients with and without symptoms of CIPN. We found no meaningful correlations between patient-reported symptoms and electrophysiological findings, and ROC curve analysis revealed a low overall accuracy of NCSs to discriminate between patients with and without symptoms indicative for CIPN, similar to the previously reported literature [16,21,22]. We assume an NCS can be normal during the early stages of axonopathy and it only monitors large-diameter fibers, while CIPN also affects components of the PNS other than large-diameter fibers, including thinly myelinated (A-delta) and unmyelinated (C) fibers [23,24], causing symptoms of dysfunction in temperature sensation, pain and autonomic dysfunction, which are not adequately measured by standard sensory nerve conduction studies. Therefore, some CIPN symptoms might be associated with small fibers, which were not measured. Our findings are in line with previous reports showing that NCSs are not appropriate for the diagnosis of CIPN [22,25]. A possible alternative for the non-invasive assessment of CIPN could be neuromuscular ultrasound [26] as ultrasound could be used to reveal nerve fascicle enlargement within the hyperechoic epineurial rim, typically seen in neuropathies [27,28]. Also, high-resolution magnetic resonance imaging (MRI) can be used to assess peripheral nerve structure [29]. Future studies should explore if these techniques can effectively distinguish CIPN-affected nerves from unaffected nerves. 

We found that patients who developed neurotoxic chemotherapy effects already had lower EORTC-QLQ-CIPN-20 scores and SNAP amplitudes (but not CMAP amplitudes) at baseline, thus possibly reflecting a ‘population at risk’ for developing CIPN. This is consistent with previous studies, as subclinical deficits in clinical sensory nerve testing have been reported before [30,31,32,33]. Sensory electrophysiological abnormalities at baseline might be related directly to the neoplasm, such as the remote paraneoplastic effect [34], and might be aggravated by the neurotoxic effects of chemotherapy. 

## 5. Limitations

Follow-up measurements were incomplete for many individuals, as NCSs are time-consuming, which restricts the willingness to participate. The inclusion of female patients only limits the generalizability of the findings, as higher sensory amplitudes and conduction velocities have been reported in females [35]. This study measured only the SNAPs in the upper extremity. 

## 6. Conclusions

In length-dependent axonal neuropathies that may result from chemotherapy, the lower extremity nerves are more severely afflicted. Hence, the evaluation of lower extremity peripheral nerves such as the sural nerve is more useful and should be considered in future studies. The discordance between subjective patient experience and NCSs suggests that an NCS is unsuitable as a requirement for the diagnosis of CIPN, as it does not reflect the impact of CIPN on patients. 

## Figures and Tables

**Table 1:** Baseline demographic and clinical characteristics of included patients.

	All	CIPN+	CIPN−	Sign.
Patients, *n* (%)	149	70 (47.0%)	79 (53.0%)	
Age (years)	54.5 (±12.6)	56.7 (±11.1)	52.5 (±13.5)	<0.05 *
Age at diagnosis (years)	52.5 (±12.4)	54.4 (±11.9)	50.9 (±11.9)	<0.05 *
BMI (kg/m^2^)	25.4 (±5.3)	26.0 (±11.9)	24.8 (±5.1)	0.120
Menopause status, *n* (%)				0.074
Premenopausal	63 (42.3%)	25 (35.7%)	38 (48.1%)	
Postmenopausal	82 (55.0%)	45 (64.3%)	37 (46.8%)	
Unknown	4 (2.6%)	0 (0.0%)	4 (5.1%)	
Cancer type, *n* (%)				0.663
Breast	121 (81.2%)	58 (82.9%)	63 (79.7%)	
Invasive ductal	113 (75.8%)	56 (80.0)	57 (72.2%)	
Invasive lobular	3 (2.0%)	1 (1.4%)	2 (2.5%)	
Others/unknown	5 (3.4%)	1 (1.4%)	4 (5.1%)	
Ovarian	23 (15.4%)	10 (14.3%)	13 (16.5%)	
Serous	19 (12.8%)	8 (11.4%)	11 (13.9%)	
Mucinous	1 (0.7%)	1 (1.4%)	0 (0.0%)	
Others/Unknown	3 (2.0%)	1 (1.4%)	2 (2.5%)	
Endometrium	3 (2.0%)	1 (1.4%)	2 (2.5%)	
Type 1	3 (100%)	1 (100%)	2 (2.5%)	
Cervix	1 (0.7%)	0 (0.0%)	1 (1.3%)	
Vulva	1 (0.7%)	1 (1.4%)	0 (0.0%)	
Chemotherapy type, *n* (%)				0.524
Neo-adjuvant	87 (58.4%)	40 (57.1%)	47 (59.5%)	
Adjuvant	49 (32.9%)	22 (31.4%)	27 (34.2%)	
Palliative	13 (8.7%)	8 (11.4%)	5 (6.3%)	
Chemotherapy protocol				0.524
Taxane monotherapy	23 (15.4%)	13 (18.6%)	10 (12.7%)	
Taxane followed by anthracycline	42 (28.2%)	17 (24.3%)	25 (31.6%)	
Antracyclin followed by taxane	31 (20.8%)	17 (24.3%)	14 (17.7%)	
Taxane + anthracycline combination	24 (16.1%)	9 (12.9%)	15 (19.0%)	
Taxane + platinum-based	29 (19.5%)	14 (20.0%)	15 (19.0%)	

Values are mean and ±standard deviations, or numbers. * *p*-value < 0.05. Abbreviations: BMI, body mass index; CIPN, chemotherapy-induced polyneuropathy. CIPN+: patients who reported an EORTC-QLQ-CIPN20 score of ≥30 at any time point during follow-up. CIPN−: patients who did not report an EORTC-QLQ-CIPN20 score of ≥30 at any time point during follow-up. Independent samples *t*-test was used to compare variables among normally distributed groups and Mann–Whitney U Test was used for non-normally distributed variables.

**Table 2:** Baseline nerve conduction studies.

	All	CIPN+	CIPN−	*p*-Value
SNAP Median (µV)	34.4 (±17.3)	29.6 (±13.0)	38.6 (±19.5)	<0.01 *
SNAP Radial (µV)	33.0 (±11.2)	30.4 (±10.5)	35.3 (±11.3)	<0.05 *
SNAP Ulnar (µV)	33.9 (±17.8)	28.7 (±13.9)	38.3 (±19.5)	<0.01 *
NCV Median (m/s)	56.0 (±7.0)	55.1 (±7.4)	56.7 (±6.5)	0.195
NCV Radial (m/s)	70.7 (±92.5)	61.1 (±5.7)	80.4 (±131.3)	0.582
NCV Ulnar (m/s)	57.2 (±6.0)	56.1 (±5.4)	58.1 (±6.4)	0.065
CMAP Median (mV)	8.1 (±2.4)	8.1 (±2.2)	8.2 (±2.6)	0.806
CMAP Ulnar (mV)	7.5 (±1.6)	7.3 (±1.7)	7.6 (±1.5)	0.234
Latency Median (ms)	3.5 (±0.5)	3.5 (±0.7)	3.4 (±0.4)	0.706
Latency Ulnar (ms)	2.5 (±0.3)	2.5 (±0.4)	2.5 (±0.3)	0.997

Values are mean and ±standard deviations. * *p*-value < 0.05. Abbreviations: CMAP, compound muscle action potential; SNAP, sensory nerve action potential; NCV, nerve conduction velocity. CIPN+: patients who reported an EORTC-QLQ-CIPN20 score of ≥30 at any time point during follow-up. CIPN−: patients who did not report an EORTC-QLQ-CIPN20 score of ≥30 at any time point during follow-up. Independent samples *t*-test was used to compare variables among two normally distributed groups and Mann–Whitney U Test was used for non-normally distributed variables.

**Table 3:** Correlation analysis of change in patient-reported symptoms and electrophysiological parameters, for all patients and for patients with CIPN at any time point.

a. Patients with CIPN			
% of Change from Baseline	EORTC-QLQ-CIPN20	*p*-Value	*n*
SNAP Ulnar	−0.324	0.030 *	45
NCV Ulnar	−0.013	0.931	47
SNAP Median	−0.299	0.042 *	47
NCV Median	0.040	0.790	47
SNAP Radial	−0.248	0.117	41
NCV Radial	−0.143	0.458	29
CMAP Median	−0.058	0.699	47
DML Median	0.050	0.740	47
CMAP Ulnar	0.070	0.641	47
DML Ulnar	0.113	0.451	47
**b. Patients without CIPN**			
**% of Change from Baseline**	**EORTC-QLQ-CIPN20**	***p*** **-Value**	***n***
SNAP Ulnar	0.022	0.888	45
NCV Ulnar	0.082	0.583	47
SNAP Median	−0.181	0.230	46
NCV Median	0.107	0.474	47
SNAP Radial	−0.087	0.578	43
NCV Radial	0.056	0.778	28
CMAP Median	−0.081	0.599	44
DML Median	−0.112	0.455	47
CMAP Ulnar	−0.166	0.264	47
DML Ulnar	−0.264	0.073	47

* *p*-value < 0.05. Abbreviations: CIPN, chemotherapy-induced polyneuropathy; CMAP, compound muscle action potential; DML, distal motor latency.

**Table 4:** ROC curve analysis for the performance of electrophysiological parameters in discriminating patients with and without symptoms of CIPN.

	Parameter	No. of Positive Cases	AUC (95% CI)	Sig.
Overall (% of change from baseline)	SNAP Ulnar (µV) (*n* = 90)	45	0.420 (0.301–0.538)	0.188
	SNAP Median (µV) (*n* = 93)	47	0.433 (0.316–0.551)	0.268
	SNAP Radial (µV) (*n* = 84)	41	0.475 (0.350–0.601)	0.697
	NCV Ulnar (m/s) (*n* = 84)	47	0.542 (0.425–0.660)	0.480
	NCV Median (m/s) (*n* = 84)	47	0.415 (0.296–0.534)	0.156
	NCV Radial (m/s) (*n* = 57)	29	0.500 (0.348–0.652)	1.000
	CMAP Median (mv) (*n* = 91)	47	0.382 (0.266–0.497)	0.052
	DML Median (ms) (*n* = 94)	47	0.544 (0.426–0.661)	0.466
	CMAP Ulnar (mV) (*n* = 94)	47	0.391 (0.277–0.506)	0.070
	DML Ulnar (ms) (*n* = 94)	47	0.572 (0.454–0.689)	0.232

Abbreviations: AUC, area under the curve; CIPN, chemotherapy-induced polyneuropathy; CMAP, compound muscle action potential; DML, distal motor latency.

## Data Availability

The original contributions presented in the study are included in the article, further inquiries can be directed to the corresponding author.
